# Development of Functional Fluorescent Molecular Probes for the Detection of Biological Substances

**DOI:** 10.3390/bios5020337

**Published:** 2015-06-18

**Authors:** Yoshio Suzuki, Kenji Yokoyama

**Affiliations:** 1 Health Research Institute, National Institute of Advanced Industrial Science and Technology (AIST), Central 6, 1-1-1 Higashi, Tsukuba 305-8566, Japan; 2 School of Bioscience and Biotechnology, Tokyo University of Technology, 1404-1 Katakura, Hachioji, Tokyo 192-0982, Japan; E-Mail: yokoyamakj@stf.teu.ac.jp

**Keywords:** molecular probes, sensors, metal ions, proteins, DNA

## Abstract

This review is confined to sensors that use fluorescence to transmit biochemical information. Fluorescence is, by far, the most frequently exploited phenomenon for chemical sensors and biosensors. Parameters that define the application of such sensors include intensity, decay time, anisotropy, quenching efficiency, and luminescence energy transfer. To achieve selective (bio)molecular recognition based on these fluorescence phenomena, various fluorescent elements such as small organic molecules, enzymes, antibodies, and oligonucleotides have been designed and synthesized over the past decades. This review describes the immense variety of fluorescent probes that have been designed for the recognitions of ions, small and large molecules, and their biological applications in terms of intracellular fluorescent imaging techniques.

## 1. Introduction

Recently, understanding the functioning of the human body at the molecular level is of major clinical significance. In particular, transmission of information in living cells has been studied by many researchers in various scientific fields. This has led to the development of a wide range of analytical reagents and tools, which allow us to gain information about intracellular dynamics [1].

**Figure 1:**
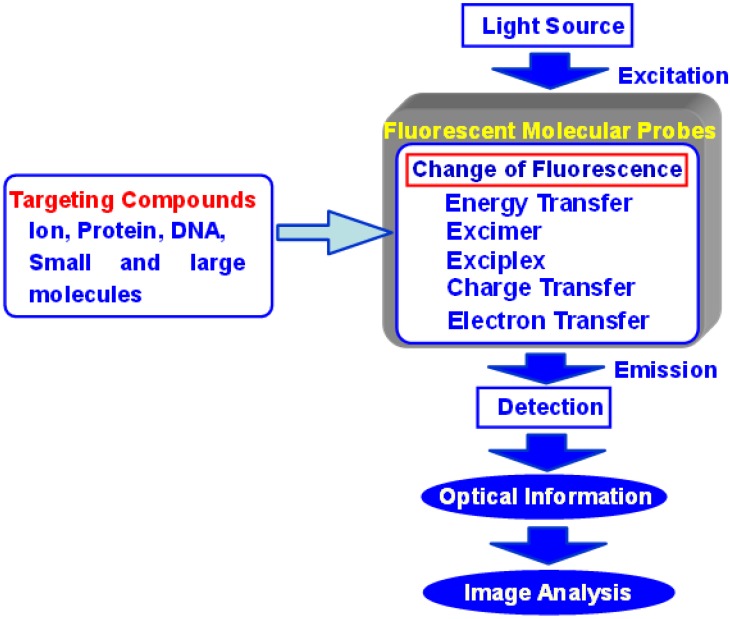
Concept of fluorescent molecular probes and their biological applications.

Several analytical methods are available for obtaining information about transmission in living cells, including absorption spectrometry, fluorescence spectrometry, electrochemical methods, chemical luminescence, and isotope-based methods. Particularly, fluorescence spectrometry is a highly sensitive and straightforward analytical method. Advances in optical instrumentation led to the development of specialized fluorescent imaging techniques such as fluorescence microscopy, confocal laser scanning microscopy, two photon excitation instruments, and evanescent excitation instruments. Recently, analytical scientists, organic chemists, and medical scientists have designed various types of fluorescent probes that emit a spectral response upon binding ions or neutral organic or inorganic molecules. These probes have enabled researchers to investigate the changes in intracellular free ions or the concentrations of molecules using fluorescent microscopy, flow cytometry, and fluorescent spectroscopy (Figure 1). Such fluorescence imaging techniques have benefited not only from the advancement of optical instruments, but also from the development of highly functional fluorescent probes based on new molecular designs. 

Several requirements need to be considered when designing a fluorescent molecular probe: the probe should display (1) efficient excitation with most laser-based instrumentation; (2) limited interference from sample autofluorescence; (3) limited cellular photodamage and scatter; (4) a high molar extinction coefficient and quantum yield, which may guarantee the use of lower dye concentrations and prevent toxicity in the living cell; and (5) presence of highly selective functional groups, which recognize the target chemical substance. Moreover, it is important to translate chemical reactions in the living body (such as structural changes in proteins, protein–protein interactions, or the formation of a duplex of DNA or RNA) to effective changes in fluorescence intensity or to a wavelength shift. For example, fluorescence resonance energy transfer (FRET) is useful to image the structural change in proteins or DNA, and the interaction of two different chemical substances [2]. FRET molecules consist of both an energy donor and an energy acceptor fluorophore. Upon excitation of the donor fluorophore, the fluorescent energy transfers from the donor to the acceptor, and fluorescence emission is observed from the acceptor fluorophore. The efficiency of FRET depends on the distance between the donor and acceptor molecule, which needs to be between 10 and 100 Å, a suitable range for the measurement of chemical interactions.

In order to detect the concentration or dynamics of a target chemical substance, the fluorescence intensity or wavelength of a probe recognizing the target is monitored. In this case, photoinduced electron transfer (PET) or photoinduced charge transfer (PCT) is useful.

In the case of PET, an electron of the highest occupied molecular orbital (HOMO) in the fluorophore is promoted to the lowest unoccupied molecular orbital (LUMO). This enables PET from the HOMO of the electron donor (serving as the potential receptor for the target of interest, and the electron donating group was indicated by “D”) to that of the fluorophore, causing fluorescence quenching of the latter [3,4,5,6]. Upon binding of the target chemical substance, the redox potential of the donor is raised so that the relevant HOMO becomes lower in energy than that of the fluorophore. Consequently, PET is no longer possible and fluorescence quenching is suppressed. In other words, the fluorescence intensity is enhanced upon binding to target compounds. In most PET sensors, the receptor involves aliphatic or aromatic amines acting as quenchers. Indeed, it is well known that PET can take place from amino groups to aromatic hydrocarbons, thus causing fluorescence quenching of the latter.

**Figure 2:**
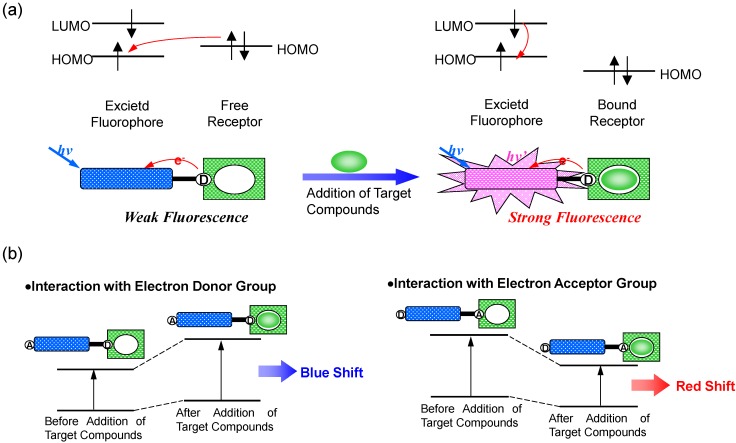
Principle of fluorescent PET (Photoinduced Electron Transfer) sensors (**a**), and spectral shift of PCT (Photoinduced Charge Transfer) sensors resulting from interaction of a bound sample with an electron-donating or electron-accepting group (**b**).

PCT, on the other hand, involves intramolecular charge transfer from the electron donor to the electron acceptor upon excitation by light when a fluorophore contains an electron-donating group (often an amino group) conjugated to an electron-accepting group [7,8,9,10]. The consequent change in dipole moment results in a Stokes shift that depends on the microenvironment of the fluorophore. Polarity probes have been designed on this basis. Thus, it can be anticipated that target substances in close interaction with the electron donor or the electron acceptor moiety will change the photophysical properties of the fluorophore, because the complexed chemical substances affects the efficiency of the intramolecular charge transfer. When an electron donor within the fluorophore interacts with a sample, the latter reduces the electron-donating character of this group. Due to the resulting reduction of conjugation, a blue shift in the absorption spectrum is expected together with a decrease in the extinction coefficient. On the other hand, a sample interacting with the electron acceptor group enhances the electron-withdrawing characteristic of this group. The absorption spectrum is thus red-shifted and the molar absorption coefficient is increased. The fluorescence spectra are in principle shifted in the same direction as those of the absorption spectra (Figure 2). 

The present review will focus on fluorescent probes based on a unique molecular design using the above mentioned photophysical properties, which target important biological substances such as Ca^2+^, Mg^2+^, Zn^2+^, proteins, DNA, and RNA, and their biological applications for intracellular fluorescent imaging techniques. 

## 2. Ca^2+^ Selective Fluorescent Sensors

The critical evaluation of the role of calcium as an intracellular messenger requires quantitative measurement of the cytosolic free Ca^2+^ concentrations and their dynamics in response to various stimuli and cellular processes [11]. Highly localized, large increases in Ca^2+^ regulate many physiological processes, including neurotransmitter release and the opening of large-conductance Ca^2+^-activated K^+^ channels causing transient outward currents. Therefore, they are particularly important to visualize and measure. Moreover, it is the apparent temporal and spatial summation of the effects of many of these localized Ca^2+^ changes that possibly lead to global Ca^2+^ elevations, which affect events such as cell shortening and force development [12].

**Figure 3:**
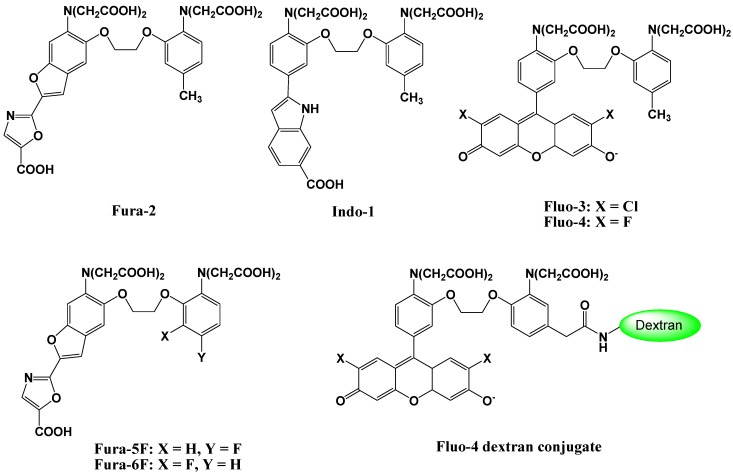
Chemical structures of Ca^2+^ fluorescent molecular probes.

Fluorescent Ca^2+^ probes, which were initially developed by Tsien *et al*. in the 1980s, have been of enormous benefit to life science and biomedical research. A new family of highly fluorescent indicators, called fura-2, has been synthesized for the biochemical studies of the physiological role of cytosolic free Ca^2+^ [13]. The chemical structure of fura-2 is shown in Figure 3. Fura-2 combines an 8-coordinate tetracarboxylate chelating site with stilbene chromophores [14]. Incorporation of the ethylenic linkage of the stilbene into a heterocyclic ring enhances the quantum efficiency and photochemical stability of the fluorophore. Compared to the widely used predecessor “quina”, fura-2 offers up to 30-fold brighter fluorescence, based on major changes in the wavelength and not just the intensity upon Ca^2+^ binding. Although it has marginally lower affinities for Ca^2+^, fura-2 has slightly longer wavelengths of excitation, and a considerably improved selectivity for Ca^2+^ over other divalent cations.

**Figure 4:**
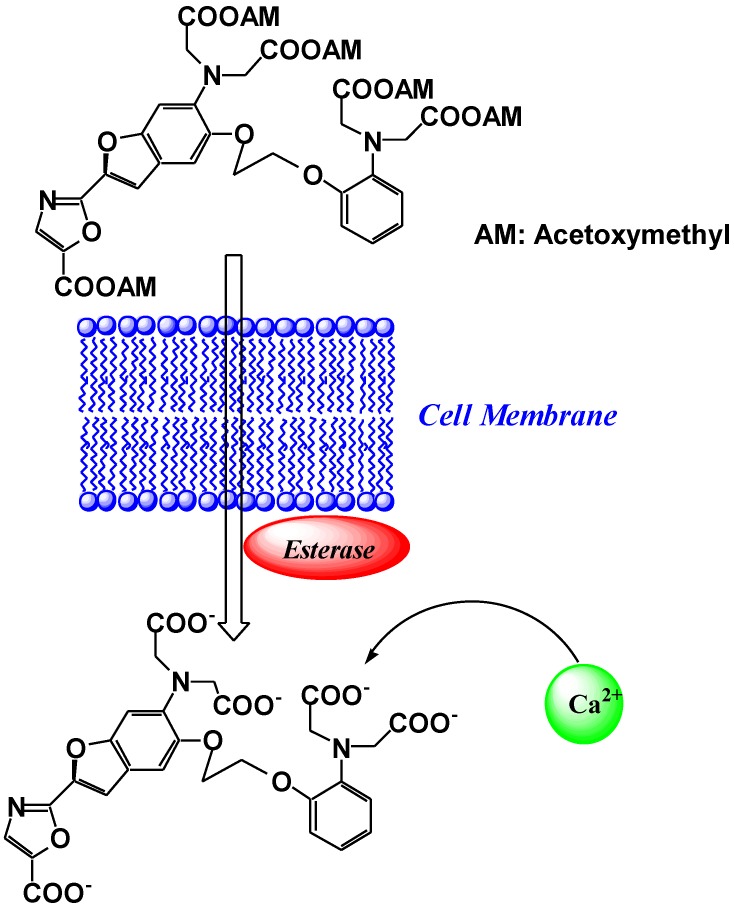
Schematic representation of the cell loading process of fura-2.

To load the fluorescent indicators into living cells, the carboxylate groups or phenolic hydroxyl groups in fura-2 are derivatized as acetoxymethyl or acetate esters, respectively. Once inside the cell, these derivatives are hydrolyzed by intracellular esterases, and indicators possessing carboxylate anions for the recognition of Ca^2+^ are released into the living cell (Figure 4) [15]. Because of these advantages, particularly the wavelength sensitivity to Ca^2+^, fura-2 is the preferred dye for many applications involving optical detection and quantification of this ubiquitous signaling ion, both inside living cells and in extracellular environments [16,17]. For example, fluorescent calcium probes are used to monitor synaptic transmissions, subcellular calcium release mechanisms in the nucleus and cytosol, and organelle-specific calcium concentration changes [18,19,20,21,22]. Since the development of fura-2, a significant effort has been made to tailor the ion-binding affinities, live cell loading, distribution properties, and wavelength dependencies for specific applications.

Visible light-excitable fluorescent indicators provide several advantages over UV light-excitable fluorescent indicators (such as fura-2 or indo-1), including (1) reduction of interference from autofluorescence of the sample; (2) reduction of cellular photodamage and light scatter; and (3) efficient excitation by most laser-based instruments (confocal laser-scanning microscopes for example).

Tsien *et al*. developed a new group of fluorescent indicators with visible excitation and emission wavelengths to measure free cytosolic Ca^2+^ [23]. Among this group, Fluo-3 and Fluo-4 (Figure 3), have gained widespread acceptance as useful general cytosolic Ca^2+^ probes because of their high Ca^2+^ affinity, large dynamic range upon Ca^2+^ binding, good cell loading properties, and excellent match of the excitation wavelength with the widely used argon ion laser [24,25]. These fluorescent indicators combine the 8-coordinate tetracarboxylate chelating site of 1,2,-bis(Z-aminophenoxyethane- N,N,N',N'-tetraacetic acid with a xanthene chromophore to produce a fluorescein-like fluorophore. These probes are essentially nonfluorescent, whereas a strong fluorescence emission is observed upon Ca^2+^ binding, and a quantum yield at the Ca^2+^ saturation of ~ 0.14 is observed. The dissociation constants for Ca^2+^ are 0.39 μM for Fluo-3, and 0.35 μM for Fluo-4, which illustrates that these indicators should give better resolution measurements of high Ca^2+^ levels than was previously possible using fura-2. The visible excitation wavelength (488 nm) of the new compounds is more convenient, not only for fluorescence microscopy and flow cytometry, but also for confocal laser scanning microscopy. Recently, imaging techniques using these probes have been extended to include the two-photon excitation technique. Moreover, Ca^2+^ imaging using these indicators has revealed the spatial dynamics of many elementary processes in Ca^2+^ signaling and has been used for cell-based high-throughput screening assays for drug discovery [26]. 

Fura-2 and Fluo-3 indicators are suitable for low Ca^2+^ concentration imaging. However, the dissociation constants of these probes are 100 nM–200 nM, which does not allow measurement of sub-micromolar Ca^2+^ concentrations. In order to solve this problem, mono- or di-fluorinated derivatives of conventional Ca^2+^ indicators, such as fura-2 and indo-1, have been designed and synthesized [27]. The chemical structures of these novel compounds (fura-5F and fura-6F) are shown in Figure 3. The fluorination, which is the addition of an electron withdrawing group, at the ortho or para position in the 1,2-bis(2-aminophenoxy)ethane-N,N,N',N'-tetraacetic acid (BAPTA) has a more pronounced weakening effect on the binding affinity than fluorination at the meta position. The dissociation constants of these probes range from 400 nM to 5.3 μM. 

A novel Fluo-4 analog, Fluo-4 dextran conjugate, has been designed and synthesized as shown in Figure 2 [28]. The attachment of a carboxamide or methylenecarboxamide moiety to the BAPTA chelator portion of Fluo-4 allows the attachment of dextrans, protein-reactive moieties, and biotin. In particular, a high affinity Fluo-4 dextran conjugate was shown to be functional in brain slices. All these new probes were spectroscopically characterized and exhibited large fluorescence increases upon calcium-binding. The biotinylated version of Fluo-4 forms a persistent complex with streptavidin, which still responds to increasing calcium concentrations with a large fluorescence increase.

**Figure 5:**
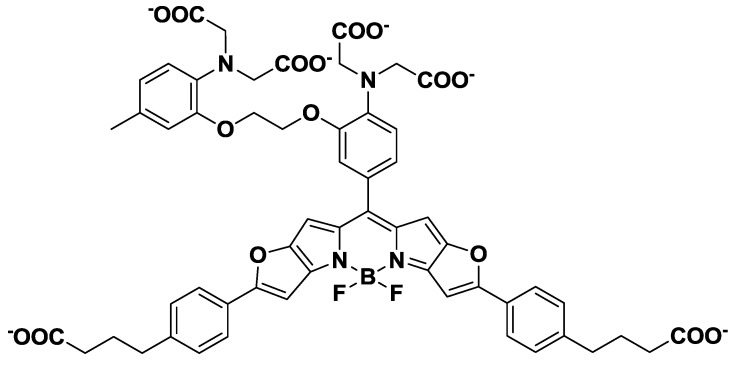
Chemical structure of KFCA.

Near-infrared (NIR) fluorescent probes, which show emission peaks from 650 nm to 900 nm, have been developed because of the minimal NIR autofluorescence from biomolecules, as well as the deep tissue penetration of NIR light. NIR calcium probes based on cyanine or squaraine chromophores have been designed and synthesized [29,30]. However, these probes have either a low quantum yield or a poor ON/OFF fluorescence signal contrast. To overcome these limitations, Suzuki *et al*. reported the development of a BODIPY-based NIR fluorescent Ca^2+^ probe (KFCA), shown in Figure 5 [31]. This probe emits sharp fluorescence spectra with high extinction coefficients, high quantum yields (emission maximum; 670 nm, Φ: 0.24), and an excellent ON/OFF ratio (120-fold), and was successfully used in real-time dual-colour intracellular Ca^2+^ imaging.

## 3. Mg^2+^ Selective Fluorescent Molecular Probes

In addition to Ca^2+^, Mg^2+^ is one of the most important divalent cations in the cell, and fluorescent probes could help increase the understanding of its many intracellular functions. Mg^2+^ plays a critical role as enzyme cofactor during DNA synthesis and protein phosphorylation. Furthermore, Mg^2+^ modulates signal transduction, various transporters and ion channels, and it is known to regulate phosphoinositide-derived second messengers. It has also been shown that Mg^2+^ concentrations change in response to chemical stimulation [32,33]. Therefore, it can be expected that changes in Mg^2+^ concentration are of physiological importance (e.g., photosynthesis, oxidative phosphorylation, and muscle contraction are modulated by Mg^2+^) [34]. Although Mg^2+^ plays such an important role in the cell, it has been much less studied than Ca^2+^.

**Figure 6:**
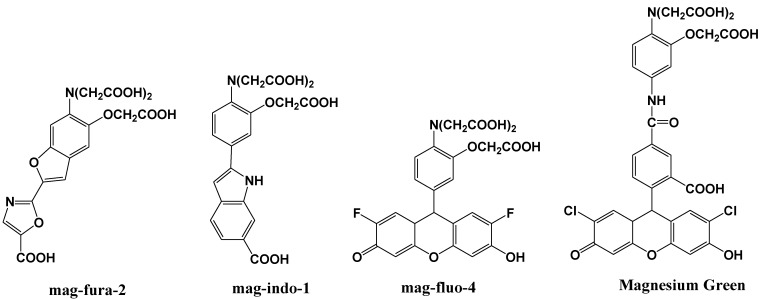
Chemical structures of magnesium fluorescent probes possessing an APTRA group.

Mag-fura-2, shown in Figure 6, is the first fluorescent magnesium indictor, developed to visualize and understand intracellular Mg^2+^ distributions [35]. The binding site for Mg^2+^ in mag-fura-2 is the APTRA (*O*-aminophenol-*N, N, O*-triacetic acid) group, and its dissociation constant for Mg^2+^ is 1.9 mM. Mag-fura-2 is excited by UV light (λ_max_: 369 nm), and shows an increase in fluorescence intensity in the visible region upon Mg^2+^ binding. Mag-Fura-2 has been used to study the intracellular Mg^2+^ concentration in cells from the liver, heart, muscle, and nervous system [36,37,38,39]. 

Thereafter, mag-indo-1 has been synthesized on the basis of the same molecular design as mag-fura-2 [40]. The chemical structure of mag-indo-1 is shown in Figure 6. It has a dissociation constant of 2.7 mM for Mg^2+^ and is excited by UV light to emit fluorescence in the visible region. Mag-fura-2 undergoes an appreciable shift in excitation wavelength upon binding to Mg^2+^, whereas mag-indo-1 shows a shift in both its excitation and emission wavelengths. The affinity of mag-indo-1 for Mg^2+^ is lower than that of mag-fura-2, which is useful for the measurement of high spikes in intracellular Mg^2+^, such as the glutamate-stimulated Mg^2+^ concentration change or the temporal analysis of Ca^2+^-induced Mg^2+^ mobilization in neurons [41,42,43].

Several other fluorescent indicators have been developed that are excited by visible light, such as Magnesium Green and mag-fluo-4, shown in Figure 6 [44,45]. Magnesium Green also possesses the APTRA group as the binding site for Mg^2+^ but shows a higher affinity for Mg^2+^ than both mag-fura-2 and mag-indo-1. Its excitation maximum is 490 nm and the emission maximum is 520 nm. Upon binding to Mg^2+^, the fluorescence intensity of Magnesium Green increases without a shift in wavelength. Magnesium Green has been used to probe intracellular Mg^2+^ for the investigation of the binding of free Mg^2+^ by the bacterial SecA protein and the detection of ATP hydrolysis in spontaneously contracting cardiomyocytes [46,47].

**Figure 7:**
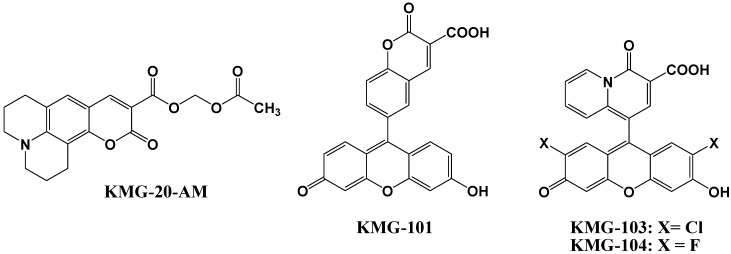
Chemical structures of magnesium fluorescent probes possessing *β*-diketone group.

Mg^2+^ fluorescent indicators possessing APTRA as Mg^2+^ binding site recognize and bind Ca^2+^ more tightly than Mg^2+^ (for example, the ratio between *K*_Mg_ and *K*_Ca_ was 0.013 in Mag-Fura-2, 0.013 in Mag-Indo-1, and 0.003 in Magnesium Green), which interferes with correct Mg^2+^ measurement and limits the interpretation of the read-out [48,49]. Therefore, Suzuki *et al*. reported the development of a novel Mg^2+^ fluorescent molecular probe KMG-20-AM (shown in Figure 7), in which AM is an acetoxymethyl group, based on a coumarin possessing a charged *β*-diketone structure [50]. This fluorescent probe produces a red shift from 425 to 445 nm in the absorption spectra after formation of a complex with Mg^2+^. The fluorescence spectrum of this probe also showed a red shift from 485 to 495 nm and an increase in fluorescence intensity after Mg^2+^ complex formation. This probe showed a “seesaw-type” fluorescent spectral change with the isosbestic point at 480 nm due to the light excitation at 445 nm, which indicates that ratiometry can be used for the measurement. The dissociation constant (*K*_d_) of KMG-20-AM was 10.0 mM. The association constants of the probe are ~3 times higher for Mg^2+^ than for Ca^2+^, and the selectivity of Mg^2+^ over Ca^2+^ is over 200 times higher than that of other Mg^2+^ fluorescent molecular probes such as mag-fura-2 and Magnesium Green. This type of probe was applied for intracellular fluorescent imaging of Mg^2+^. After the addition of KMG-20-AM into PC12 cells, strong fluorescence was observed in the cytoplasm and weak fluorescence in the nuclear region. After treatment with high-K^+^ medium, the fluorescence intensity increased due to increasing intracellular Mg^2+^ concentrations. The Mg^2+^ release from intracellular stores was successfully imaged using this Mg^2+^ fluorescent probe. As another application of KMG-20-AM, Kopelman *et al*. developed intracellular Mg^2+^-sensitive nanoparticles using PEBBLEs [51].

Komatsu *et al*. reported the design, synthesis, and cellular application of three novel Mg^2+^ fluorescent probes, KMG-101, -103, and -104, shown in Figure 7 [52]. Like KMG-20-AM, these compounds possess a charged *β*-diketone as a binding site specific for Mg^2+^, and a fluorescein residue as the fluorophore that can be excited with an Ar^+^ laser, widely used in confocal scanning microscopy. This molecular design leads to an intensive off-on-type fluorescent response toward Mg^2+^ ions. The two fluorescent probes KMG-103 and -104 show suitable dissociation constants (*K*_d,Mg^2+^_ = 2 mM) and nearly a 10-fold fluorescence enhancement over the intracellular magnesium ion concentration range (0.1 mM to 6 mM), allowing high-contrast, sensitive, and selective Mg^2+^ measurements. For intracellular applications, the membrane-permeable probe KMG-104-AM was synthesized and successfully incorporated into PC12 cells. The increase in free Mg^2+^ upon application of the mitochondrial uncoupler FCCP could be followed over time. By using a confocal microscope, the intracellular 3D Mg^2+^ distributions were successfully observed with the KMG-104-AM probe. 

**Figure 8:**
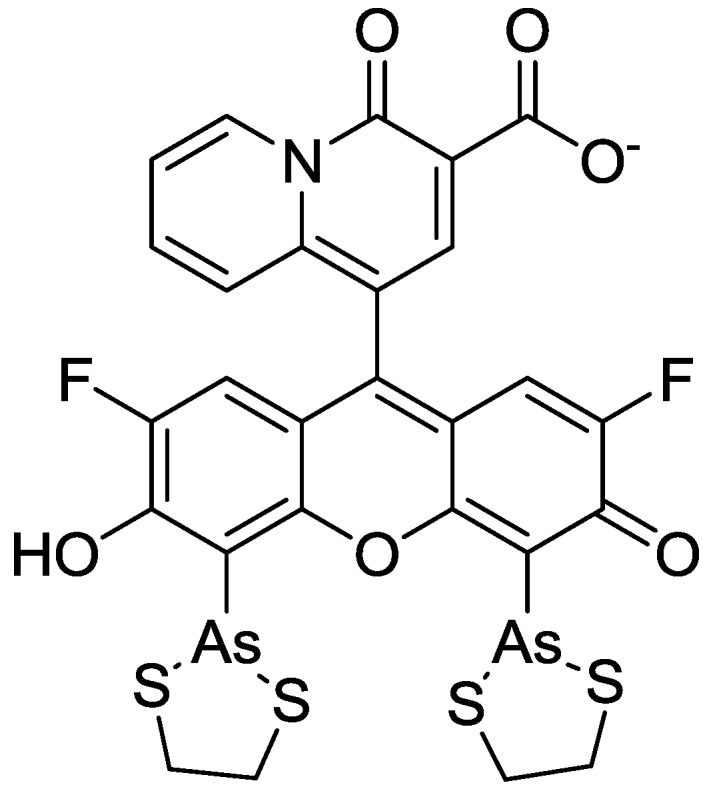
Chemical structure of KMG-104-AsH.

In order to incorporate the fluorescent probe KMG-104 into a protein and to study the mobilization and underlying mechanisms of Mg^2+^, KMG-104-AsH has been developed (shown in Figure 8). KMG-104-AsH is composed of the highly selective fluorescent Mg^2+^ probe and a tetracysteine peptide tag (TCtag), which can be genetically incorporated into any protein [53]. The fluorescence intensity of KMG-104-AsH increased by more than 10-fold by binding to both the TCtag peptide and Mg^2+^, and had a highly selective affinity for Mg^2+^ (K_d_ for Mg = 1.7 mM, K_d_ for Ca = 100 mM). Moreover, fluorescent imaging of intracellular Mg^2+^ in HeLa cells showed that this FlAsH-type Mg^2+^ sensing probe was membrane-permeable and bound specifically to tagged proteins, such as TCtag-actin and mKeima-TCtag targeted to the cytoplasm and the mitochondrial intermembrane space. This probe is expected to be a valuable tool for elucidating the dynamics and mechanisms of intracellular localization of Mg^2+^.

## 4. Zn^2+^ Selective Fluorescent Molecular Probes

The essential role of Zn^2+^ in human metabolism and the biochemical processes in which Zn^2+^ takes part are well documented [54,55]. Approximately 300 enzymes contain Zn^2+^, either for structural purposes or as part of a catalytic site, with the majority of this Zn^2+^ being tightly bound. In addition to this tightly bound Zn^2+^, there exists a readily exchangeable pool of less firmly bound Zn^2+^, termed available Zn^2+^, which comprises approximately 10% of the total cellular Zn^2+^. The latter pool is important in various processes associated with cell activation and growth, including apoptosis, gene expression, neurotransmission, signal transduction, and enzyme regulation. In addition to modulating neuronal transmission, Zn^2+^ is reported to contribute to neuronal injury under certain acute conditions, to suppress or induce apoptosis, and to induce the formation of *α*-amyloid, which is related to the etiology of Alzheimer’s disease [56,57,58].

**Figure 9:**
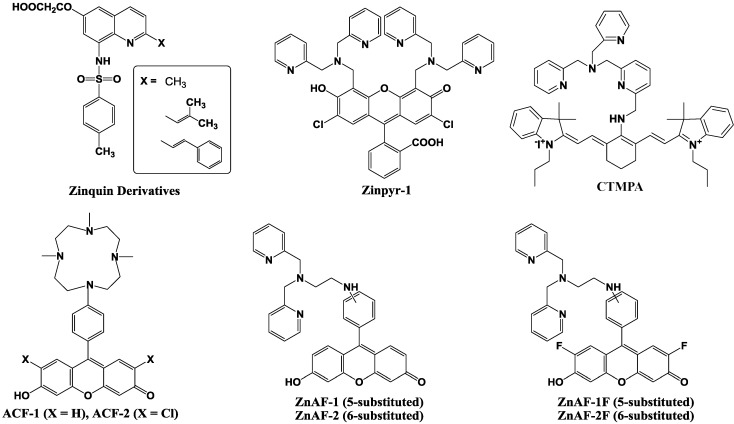
Chemical structures of zinc fluorescent probes.

The initial Zn^2+^ fluorescent indicators, such as Zinquin, were based on an 8-*p*-toluenesulfonamidoquinoline derivative shown in Figure 9 [59,60]. Zinquin ester readily traverses cell membranes and is thought to be hydrolyzed by cellular esterases in the cytoplasm. The resulting acid is deprotonated at intracellular pH, and the charged molecule has a decreased ability to cross the cell membrane and escape from the cell. Zinquin ester has been shown to be effective in the detection of zinc(II) in a variety of mammalian cells [61,62,63].

Next, 2-substituted Zinquin derivatives were designed and synthesized [64]. Compounds with isobutyl, isobutenyl, and styryl side chains (shown in Figure 9), exhibit an increased fluorescence intensity compared to that of Zinquin in the presence of Zn^2+^ by longer excitation wavelengths. However, the quinoline-based probes require UV excitation (around 350 nm), which can be damaging to cells, and have a relatively low fluorescence with quantum yields ≈ 0.1 and molar extinction coefficients ≈ 10 × 10^3^ M^−1^ cm^−1^.

To achieve high-affinity binding without quinoline sulfonamide or EGTA-based chelating moieties, the bis(2-pyridylmethyl)amine (di-2-picolylamine or DPA) moiety was selected and combined with fluorescein as reporter group because of its large extinction coefficient, high quantum yield, membrane permeability, and the availability of the optical filter sets for fluorescence microscopy. The resulting probe is called Zinpyr-1 and its chemical structure is shown in Figure 9 [65]. The dissociation constant of Zinpyr-1 is ~ 1 nM at pH 7 and it has essentially no measurable affinity for Ca^2+^ or Mg^2+^. Zinpyr-1 has an excitation maximum at 515 nm and a fluorescence quantum yield of 0.39 in the absence of Zn^2+^. With saturating Zn^2+^ concentrations (25 *μ*M), the excitation maximum shifts to 507 nm, and the quantum yield increases to 0.87. Furthermore, Zinpyr-1 is not expected to present a challenge to membrane permeability, because of its structural similarity to the membrane-permeable heavy metal chelator *N*,*N*,*N*′ ,*N*′ -tetra(2-picolyl)ethylenediamine (TPEN). Zinpyr-1 has been shown to be loaded in Cos-7 cells where it responds to changes in Zn^2+^ concentration [66,67].

Zinpyr-1 strongly fluoresces upon Zn^2+^ addition to cells. However, its disadvantage is that its basal fluorescence is high (quantum yield: 0.39) and that it is pH-sensitive with a p*K*a of 8.3. Thus, the fluorescence can be changed by intracellular pH changes under physiological conditions, and such pH changes are observed in many cells exposed to certain biological stimuli.

Nagano *et al*. have therefore developed two new probes for Zn^2+^, 6-hydroxy-9-[4-(4,7,10-trimethyl-1,4,- 7,10-tetraazacyclododecan-1-yl)]- phenyl-3H-xanthen-3-one (ACF-1; azacrownfluorone) and its 2,7-dichloride derivative (ACF-2), shown in Figure 9 [68]. The fluorophore of ACF-1 is a fluorescein derivative, 6-hydroxy-9-phenylfluorone. This fluorophore is directly linked to a macrocyclic polyamine, which strongly complexes with transition metals such as Zn^2+^ and Cu^2+^. At pH 10, the fluorescent intensity of their 9-(1,4,7,10-tetraazacyclododecyl) methylanthracene increased 14-fold upon Zn^2+^ addition. However, under neutral conditions, the nitrogen atom in this molecule, which is linked to the anthracene moiety through a methylene bridge, is protonated and the fluorescent intensity is increased without the addition of Zn^2+^. This is due to the elimination of PET upon protonation. To avoid this, the macrocyclic polyamine was bound directly to the fluorophore in the design of our ACFs. The nitrogen atom in the fluorophore results in lower p*K*a values, and consequently, the fluorescent intensity of the ACFs at pH 7 was comparable to that at around pH 10. The ACF-Zn^2+^ complex thereby fluoresces at a physiological pH. 

However, further improvements in ACF-1 and ACF-2 are desirable in two respects, *i.e*., the slow complex formation rate and the small quantum yield. ACFs require about 100 min for completion of the complex formation due to the properties of the macrocyclic polyamine ring, which is the acceptor of Zn^2+^. To overcome these demerits, ZnAF-1 and ZnAF-2, utilizing N,N,N',N'-tetrakis(2-pyridylmethyl)ethylenediamine (TPEN) as the acceptor of Zn^2+^, have been developed (Figure 9) [69]. Fluorescein was employed as a fluorophore instead of 6-hydroxy-9-phenylfluorone, the fluorophore of ACFs, because of its larger quantum yield. Upon addition of Zn^2+^, the fluorescence intensity increased by 17-fold for ZnAF-1 and 51-fold for ZnAF-2 at pH 7.5. At this pH, the fluorescence intensity of ZnAF-1 or ZnAF-2 itself is very low. The quantum yield is only 0.02 for both ZnAFs, and is not increased by pH change. However, the fluorescence intensity of the Zn^2+^ complex with ZnAF-1 or ZnAF-2 is decreased below pH 7.0. Therefore, although ZnAF-1 and ZnAF-2 are useful above pH 7.0, the signal is affected, for example, under conditions of acidosis.

New fluorescent probes for Zn^2+^, ZnAF-1F and ZnAF-2F, have been developed, whose chemical structures are described in Figure 9 [70]. Fluorescein is again used as a fluorophore, and N,N-bis(2-pyridylmethyl)ethylenediamine is used as the binding site for Zn^2+^. The quantum yields of ZnAF-1F and ZnAf-2F are 0.004 and 0.006, respectively, under physiological conditions (pH 7.4) due to the PET mechanism. Upon addition of Zn^2+^, the fluorescence intensity is quickly increased up to 69-fold for ZnAF-1F and 60-fold for ZnAF-2F. The apparent dissociation constants (*K*_d_) are in the nanomolar range, which affords a sufficient sensitivity for biological applications. ZnAFs do not fluoresce in the presence of other biologically important cations such as Ca^2+^ and Mg^2+^, and are insensitive to a change in pH. The complexes formed between Zn^2+^ and ZnAF-1 or ZnAF-2 decrease in fluorescence intensity below pH 7.0 due to protonation of the phenolic hydroxyl group of fluorescein, whose p*K*a value is 6.2. On the other hand, the Zn^2+^ complexes of ZnAF-1F and ZnAF-2F emit stable fluorescence under neutral and slightly acidic conditions because the p*K*a values are shifted to 4.9 by substitution of the electron-withdrawing fluorine at the ortho-position of the phenolic hydroxyl group. A diacetyl derivative of ZnAF-2F, ZnAF-2F DA, was synthesized for application in living cells. ZnAF-2F DA can penetrate the cell membrane, and is hydrolyzed by esterase in the cytosol to yield ZnAF-2F, which is retained in the cell [71,72].

Guo *et al*. reported the cyanine-based fluorescent probe, CTMPA (Figure 9), which was synthesized from 2,6-bis(hydroxymethyl)pyridine as starting material [73]. The emission maximum of CTMPA is at 730 nm, whereas that of the CTMPA-Zn^2+^ complex is around 590 nm, which enables successful ratiometic measurement of Zn^2+^ concentrations. CMTPA was successfully used to detect Zn^2+^ release during cellular apoptosis, endogenous Zn^2+^ in living zebrafish, as well as the Zn^2+^ in neuromasts of zebrafish. 

## 5. Protein Selective Detection Using Fluorescent Probes

**Figure 10:**
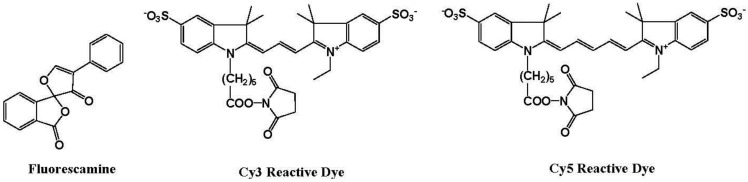
Chemical structures of fluorescent molecular probes for the labeling of proteins.

Biochemists need various ways to detect proteins with a high sensitivity and good binding linearity to facilitate both qualitative and quantitative analysis. Several fluorescent reagents have been developed for the detection of proteins in solution, such as fluorescamine and cyanine dyes (Figure 10) [74]. Fluorescamine does not produce fluorescence emission, but a strong green fluorescence at 495 nm is observed when excited at 395 nm after reaction with the primary amine in the protein.

Hydrophobic cyanine dyes indicate an increase in the emission intensities upon binding to protein-sodium dodecyl sulfate (SDS) complexes. However, protein measurement using these reagents in several analytical methods, including fluorescence microscopy, has some disadvantages, including (i) long reaction time; (ii) aggregation of dyes; (iii) small Stokes shift; and (iv) non-linear and sigmoidal calibration curves. 

**Figure 11:**
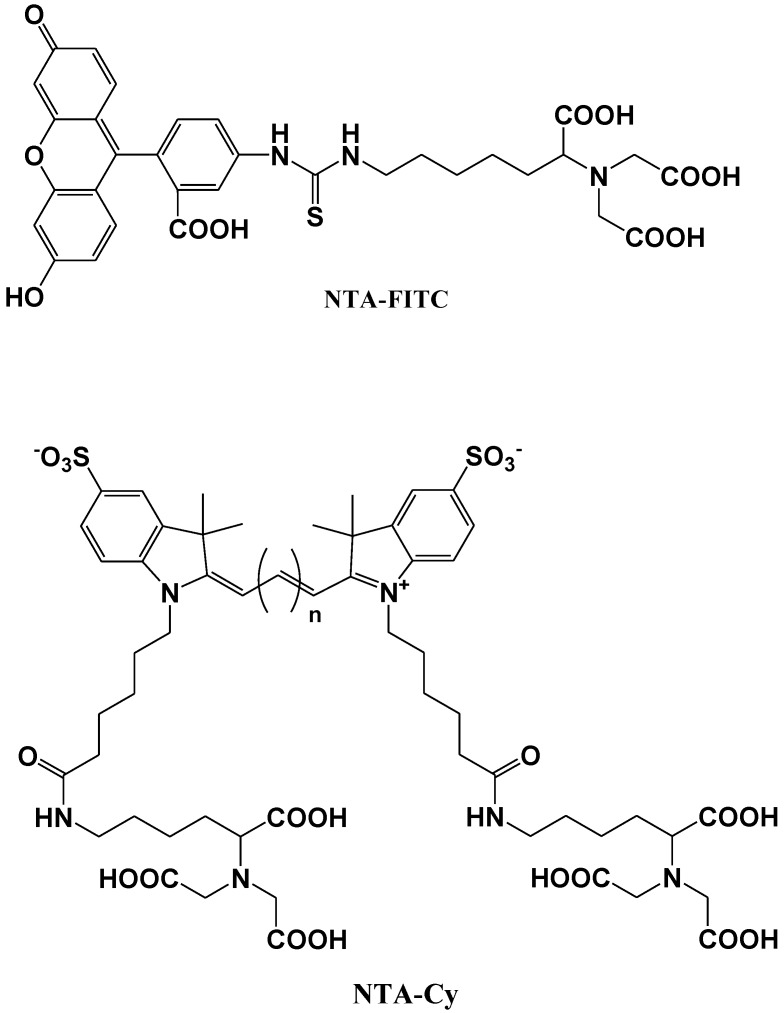
Chemical structures of fluorescent molecular probes for the labeling of histidine in proteins.

Katayama *et al*. reported the development of a fluorescent probe (NTA-FITC), which contains a nitrilo triacetic acid (NTA) moiety. The Ni^2+^ ion in this moiety acts as a binding site for an oligo-histidine sequence, and can therefore lead to the site-specific interaction of the fluorescent probe with the tagged protein (Figure 11) [75]. The NTA group forms a sandwich-type metal complex together with the oligohistidine (his-tag). This ‘his-tag’ is often attached to proteins for convenient purification, but could also be used as a tool to detect a peptide or protein on a nitrocellulose membrane using a novel fluorescent NTA derivative.

Kapanidis *et al*. reported that the hexahistidine tag should tightly interact with the (Ni^2+^:NTA) *n*-fluorochrome conjugates and thus should be able to mediate site-specific fluorescent labeling [76]. A new probe (NTA-Cy) consists of the widely used cyanine fluorochromes Cy3 and Cy5 having one pendant Ni^2+^:NTA-moiety, (Ni^2+^:NTA)_1_-Cy3 and (Ni^2+^:NTA)_1_-Cy5, or two pendant Ni^2+^:NTA- moieties, (Ni^2+^:NTA)_2_-Cy3 and (Ni^2+^:NTA)_2_-Cy5 (Figure 11). These probes all have the advantage that they are (i) compatible with widely used hexahistidine-tag-based protein-purification and protein-immobilization systems; (ii) applicable to a large library of existing hexahistidine-tagged proteins; and suitable (iii) for labeling of N-termini, C-termini, and internal sites; (iv) for *in situ* labeling; and (v) for use with cyanine fluorochromes having different spectroscopic and photophysical properties (Cy3 and Cy5, probably also Cy3.5, Cy5.5, and Cy7).

**Figure 12:**
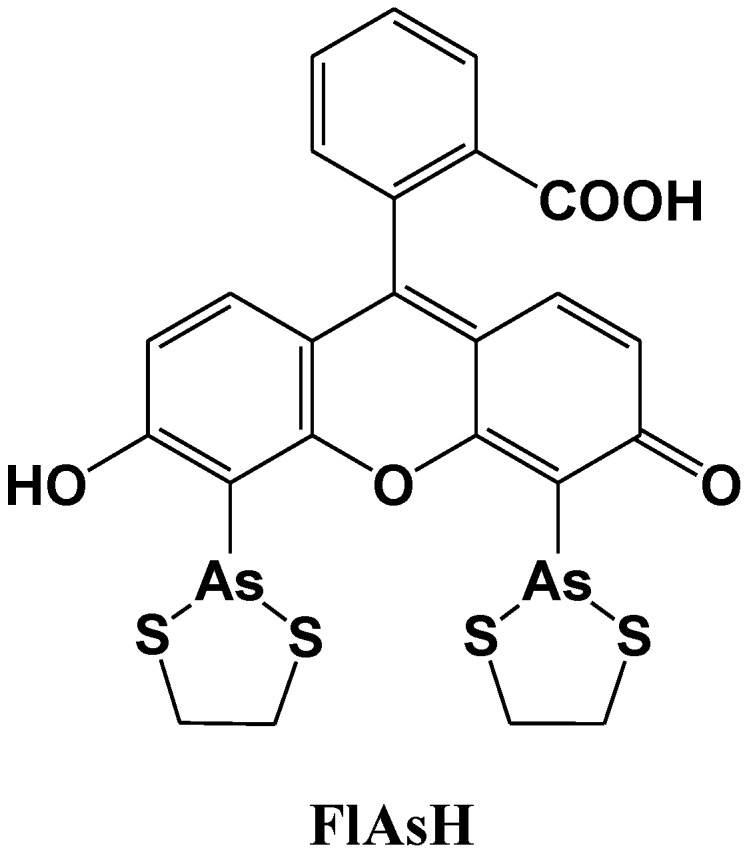
Chemical structure of a fluorescent molecular probe for the labeling of cysteine in proteins.

Tsien *et al*. reported a site-specific fluorescent labeling reagent for recombinant proteins in living cells (Figure 12) [77]. The sequence Cys-Cys-Xaa-Xaa-Cys-Cys, where Xaa is a noncysteine amino acid, is genetically fused to or inserted within the protein, where it can be specifically recognized by FlAsH. FlAsH is a membrane-permeant fluorescein derivative with two As(III) substituents, which fluoresces only after the arsenics bind to the cysteine thiols in the target sequence. The *in vitro* affinities and detection limits in living cells are optimized with Xaa-Xaa = Pro-Gly, suggesting that the preferred peptide conformation is a hairpin rather than the previously proposed R-helix. Many analogues of FlAsH have now been synthesized, including ReAsH, a resorufin derivative which is excitable at 590 nm and red fluorescent. These analogous biarsenicals enable affinity chromatography, fluorescence anisotropy measurements, and electron-microscopic localization of tetracysteine-tagged proteins.

**Figure 13:**
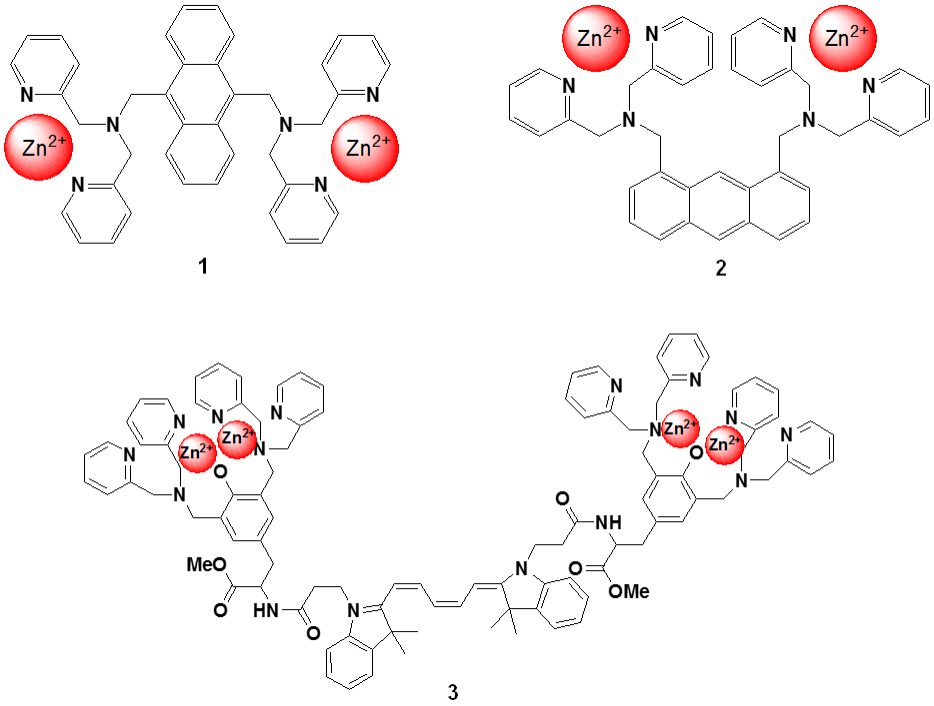
Chemical structures of fluorescent molecular probes targeting phosphorylated peptide or proteins.

Hamachi *et al*. described fluorescent probes (compound 1 and 2 in Figure 13) that target phosphorylated groups in peptides or proteins [78]. These probes consist of two anthracene derivatives as fluorophores, and a Zn^2+^ selective dipicolylamine group that recognizes the phosphorylated chemical species. The fluorescent spectral change of the probe takes place upon binding to the phosphorylated groups. It is clear that these chemosensors can detect a consensus peptide sequence phosphorylated by v-Src with a high affinity (10^7^ M^−1^) in aqueous solution. To take advantage of this phenomenon, a new peptide tag/artificial probe pair, DpaTyr/D4, composed of a genetically encodable oligo-aspartate sequence and the corresponding multinuclear Zn^2+^ complexes (compound 3 in Figure 13) were designed and synthesized [79]. The strong binding affinity of the Zn^2+^-DpaTyr probes with the D4-tag is a result of the multiple coordination bonds and the multivalent effect. It was quantitatively measured by isothermal titration calorimetry. The high affinity between the tag and the probe, indispensable for selective protein labeling, enabled the pair to be used for the labeling and fluorescence imaging of a membrane-bound receptor protein in an intact cell configuration without significantly affecting the receptor signal transduction. Based on the above molecular design, various fluorescent probes for the selective protein recognition have been developed [80,81].

Kinoshita *et al*. developed a zinc complex, (1,3-bis[bis(pyridin-2-ylmethyl)amino]propan-2-olato dizinc(II) complex), also called “Phos-tag”, for the detection of phosphoproteins, as well as a fluorescence resonance energy transfer (FRET) system for the analysis of the dephosphorylation of a 5-carboxyfluorescein (FAM)-labeled phosphopeptide by using a Phos-tag derivative attached to a (7-amino-4-methylcoumarin-3-yl)acetic acid (AMCA) moiety [82]. This FRET system is based on the principle that the Phos-tag captures the phosphopeptide in preference to its nonphosphorylated counterpart. The association between the phosphopeptide and the Phos-tag molecule brings the donor AMCA (excitation: 345 nm) close to the acceptor FAM (emission: 520 nm), resulting in an efficient FRET signal. Phosphorylation of a FAM-labeled peptide substrate by a kinase, such as the epidermal growth factor receptor or c-Src, and the dose-dependent inhibitions of the kinase reactions could be determined from real-time changes in the FRET efficiency.

**Figure 14:**
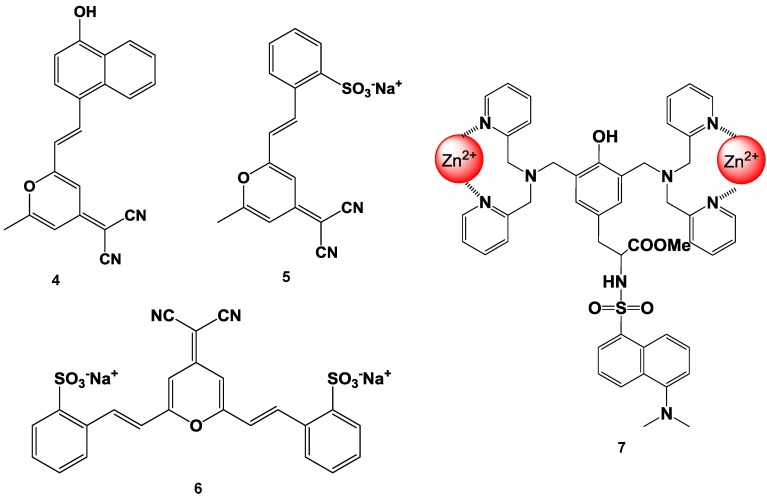
Chemical structure of fluorescent probe for the detection of protein.

Suzuki *et al*. reported fluorescent molecular probes possessing cyanopyranyl moieties or zinc (II) complexes that were designed and synthesized to detect proteins via non-specific binding (compounds 4, 5, 6, and 7 in Figure 14) [83,84,85,86]. These fluorescent probes did not produce any fluorescent emission in the absence of protein. On the other hand, the fluorescence spectra of these probes showed a large Stokes shift and dramatic increase in intensity after the addition of BSA. The fluorescence intensities of the probes were plotted as a function of the protein concentration. A good linear relationship was observed for up to 1 mg/mL of protein and the detection limit was found to be 100 ng/mL at the given assay conditions. Similar results were observed for the measurements of not only BSA, but also other proteins (for example BGG). The responses of these probes were not affected by the presence of various non-protein substances (such as inorganic salts and chelating agents). Moreover, high performance staining for 1D and 2D SDS-PAGE was carried out using a novel protein-binding fluorophore. In order to achieve the high-throughput analysis of proteins for SDS-PAGE, the general protocols for in-gel protein staining (SDS-PAGE, fixation, staining, washing, and detection) were simplified in order to produce a straightforward and rapid protocol. As a result, protein staining could be performed in a minimum of only 30 min in this study. The protein-to-protein variation was low, and the detection limit was 0.4 ng/band of BSA (signal-to-noise ratio was 3.0), which was as sensitive as the short-protocol silver staining methods.

**Figure 15:**
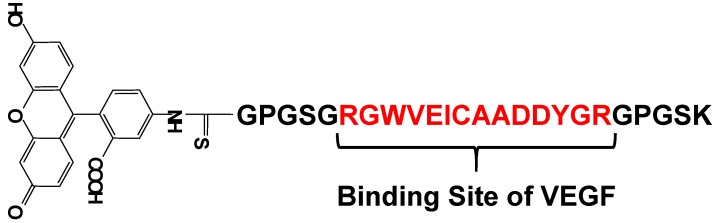
Chemical structure of a fluorescent probe for the detection of VEGF.

Vascular endothelial growth factor (VEGF) is an important regulator of angiogenesis, and it promotes the migration and proliferation of endothelial cells and the formation of new blood vessels from preexisting capillaries. Biological responses to VEGF expression result from the binding of VEGF to two membrane-embedded receptors and the subsequent intracellular signaling induced by receptor activation. Therefore, it is important to develop a tool for straightforward, rapid, and highly sensitive detection of VEGF. Suzuki *et al*. have developed a fluorescent peptide (shown in Figure 15) that emits weak fluorescence in the absence of VEGF, but strong fluorescence upon binding to VEGF [85,86]. The fluorescence intensities of the reagent were plotted in function of the VEGF concentration and a good linear relationship was observed (r^2^ > 0.984). This reagent was immobilized on an Au plate and nanopillar substrate, which was coated with a self-assembled monolayer (SAM) via covalent bonding under optimum conditions. The detection limit of VEGF in this study was 1.5 ng/mL, which is sufficient for clinical use. Using this plate and fluorescence spectrometry, VEGF could be successfully detected in rat serum. The experimental results clearly showed that this reagent is a good VEGF indicator with wide applicability.

## 6. Fluorescent Probes for the Measurement of DNA and RNA

The mutual recognition of two complementary nucleic acid strands is the molecular basis for the current approaches in oligonucleotide-based diagnostics and therapy. Much work has been devoted to the development of methods that allow for the detection of hybridization events. The hybridization assays that are most commonly used employ a solid phase in order to facilitate the separation of the bound from unbound analytes [87].

**Figure 16:**
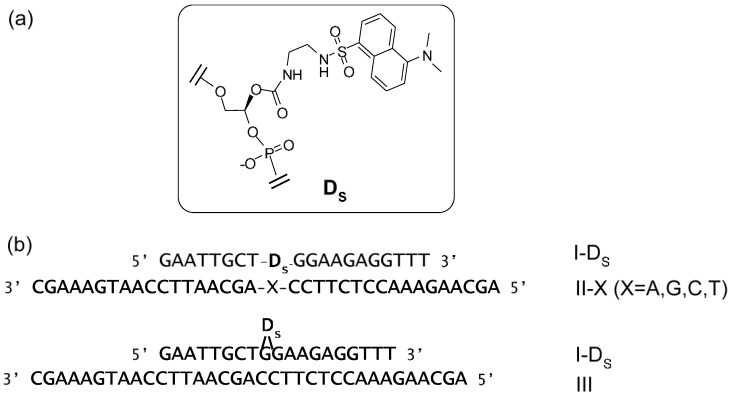
(**a**) Structure of the dansyl-unit (D) in oligonucleotide; (**b**) Sequences of ONTs. Internally dansyl-labeled ONTs (I-D_s_) has a complementary sequence with II-X and III. X in II-X indicates dA, dG, dC, or T.

Suzuki *et al*. have synthesized a nonnucleoside amidite block of dansyl fluorophore to prepare dansyl-modified oligonucleotides (ONTs) (Figure 16) [88]. The fluorescence intensities of dansyl-ONTs were specifically increased by the presence of adjacent guanosine residues but significantly reduced in a dansyl-flipping duplex. These changes were caused by a solvatochromism effect due to the number of guanines, which are hydrophobic functional groups, and the external environment of the dansyl group. The fluorescence intensities could be plotted as a function of the ONT concentrations and the increase in the fluorescence was observed to equimolar concentrations of target DNA. This duplex exhibited a higher melting temperature relative to the corresponding duplexes containing other base pairs. Similar changes in fluorescence could be detected upon hybridization of the ONTs with complementary RNAs. 

Ito *et al*. reported a new nucleic acid-based fluorescence probe based on an aptamer divided into two probes, which are then attached with a chemically reactive fluorogenic compound [89]. The protein-dependent association of the two probes accelerates a reduction-triggered fluorogenic reaction and indicates the presence of the target protein, which is detected by the monitoring of fluorescence enhancement. The fluorescence signal is caused by the deprotection of the azidomethyl group in fluorescein. Fluorescence emission is detected at 522 nm and enhanced by about 20-fold in the presence of the target peptide. An oligonucleotide-based reduction-triggered fluorescence probe was successfully applied for the detection of the Rev peptide in solution.

**Figure 17:**
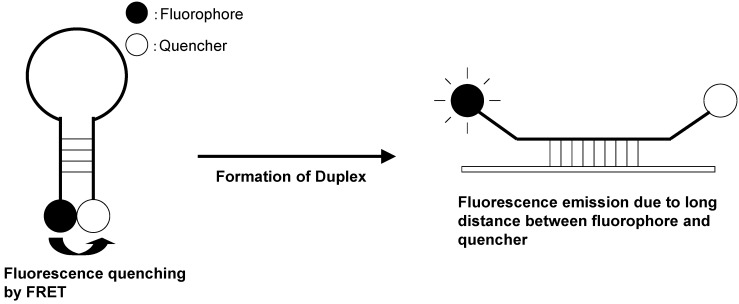
Schematic representation of molecular beacon.

Molecular beacons are hybridization probes that can report the presence of complementary nucleic acid targets without having to separate probe–target hybrids from excess probes in the hybridization assay (Figure 17) [90,91]. Because of this property, they have been used for the detection of RNAs within living cells, to monitor the synthesis of specific nucleic acids in sealed reaction vessels, and for the construction of self-reporting oligonucleotide arrays [92,93]. They can be used to perform homogeneous one-tube assays for the identification of single-nucleotide variations in DNA and for the detection of pathogens. A molecular beacon probe is a hairpin-shaped, single-stranded ONT comprised of a probe sequence embedded within complementary sequences that form a hairpin stem. A fluorophore is covalently attached to one end of the oligonucleotide, and a nonfluorescent quencher is covalently attached to the other end. In the absence of a target, the stem of the hairpin holds the fluorophore so close to the quencher that fluorescence does not occur. When the probe binds to its target, the rigidity of the probe–target duplex forces the stem to unwind, causing the separation of the fluorophore and the quencher and restoration of fluorescence. This permits the detection of probe–target hybrids in the presence of unhybridized probes.

Darby *et al*. reported the development of molecular beacons to detect the melting profiles for intramolecular DNA duplexes, triplexes, and quadruplexes [94]. These molecular beacons have both a fluorophore (fluorescein) and a fluorescence quencher (methyl red) attached either to the deoxyribose or to the 5′ position of dU. When the DNA structure is folded, the fluorophore and quencher are close, and fluorescence emission is quenched. On the other hand, when the DNA structure melts, the fluorophore and quencher are separated from each other, and fluorescence emission is observed. To take advantage of this technique, the stability of triplexes containing different base analogues is assessed, and the selectivity of a triplex-binding ligand for the triplex rather than the duplex is confirmed. 

**Figure 18:**
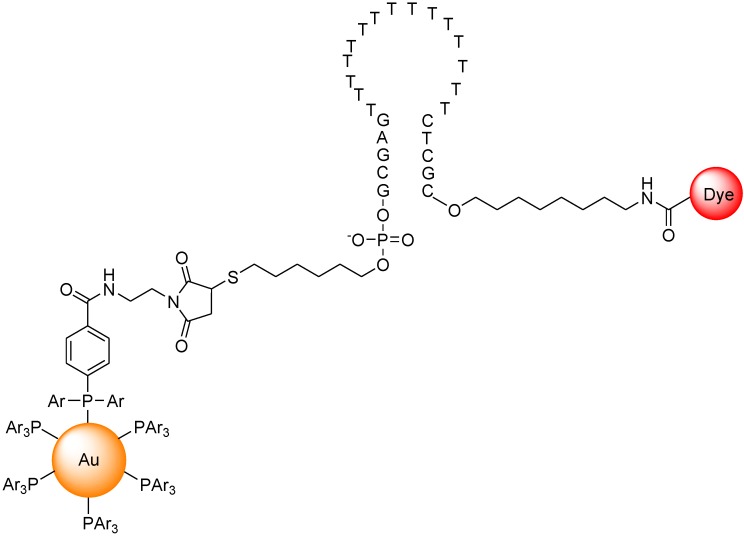
Chemical structure of molecular beacon based on gold nano particle.

Dubertret *et al*. reported a molecular beacon with a fluorophore and a 1.4 nm diameter gold nanoparticle as a quencher (Figure 18) [95]. The fluorescence quenching efficiency depends on the distance between the fluorophore and the gold nanoparticle. The fluorescence of this hybrid molecule increases by a factor of as much as several thousand as it binds to a complementary single-stranded DNA. This composite molecule is a different type of conventional molecular beacon with a sensitivity enhanced up to 100-fold. In competitive hybridization assays, the ability to detect a single mismatch is eightfold greater with this probe than with other molecular beacons.

Various types of molecular beacons have been developed, and their characteristics have been summarized in other reviews [96,97,98].

## 7. Conclusions

The present review illustrates the immense variety of fluorescent probes that have been designed for the recognitions of cations and large molecules such as proteins and DNA. The emphasis is placed on the understanding of the photophysical changes induced by target chemical substances, which should help both the user and the designer of this type of sensors. In the future, bioimaging techniques will be used not only for basic research in cellular biology, but also for the mechanistic analysis of therapeutics, and analysis of various disease mechanisms. In order to reach these objectives, it is necessary to develop new fluorescent molecular probes for each unique application. The combination of advanced techniques from various different scientific fields (medicine, biology, analytical chemistry, organic chemistry, materials science, and even some branches of engineering) will make it possible to create functional materials for the analysis of the dynamics and information transmission in living cells and the organization of the human body.
